# Clinical study of different doses of atorvastatin combined with febuxostat in patients with gout and carotid atherosclerosis

**DOI:** 10.12669/pjms.36.6.2945

**Published:** 2020

**Authors:** Zheng Zhang, Ming-hua Xu, Feng-ju Wei, Li-na Shang

**Affiliations:** 1 Zheng Zhang, Department of Rheumatology and Immunology, Affiliated Hospital of Hebei University, Baoding, Hebei 071000, P. R. China; 2 Ming-hua Xu, Department of Rheumatology and Immunology, Affiliated Hospital of Hebei University, Baoding, Hebei 071000, P. R. China; 3 Feng-ju Wei, Department of Traditional Chinese Medicine Department, Affiliated Hospital of Hebei University, Baoding, Hebei 071000, P. R. China; 4 Li-na Shang, Department of Ultrasound, Affiliated Hospital of Hebei University, Baoding, Hebei 071000, P. R. China

**Keywords:** Gout, Carotid atherosclerosis, Atorvastatin, Febuxostat

## Abstract

**Objective:**

To evaluate the efficacy of atorvastatin combined with febuxostat in the treatment of gout patients with carotid atherosclerosis and to observe the effects on serum tumor necrosis factor-α (TNF-α), interleukin-1β (IL-1β), and C-reactive protein (CRP) levels, carotid plaques, and the adverse reactions.

**Methods:**

Seventy patients with gout and carotid atherosclerosis admitted to Affiliated Hospital of Hebei University from January 2014 to June 2017 were randomly divided into a treatment group and a control group. The treatment group received oral febuxostat 40 mg/day combined with atorvastatin 40 mg/day. The control group was given 40 mg/day febuxostat combined with 20 mg/day atorvastatin for 90 days. The effects of treatment on TNF-α, IL-1β, and CRP levels and carotid plaques of the patients were observed.

**Results:**

After 90 days of treatment, serum TNF-α, IL-1β, and CRP levels, as well as HUA and total cholesterol (TC), decreased in both groups after treatment. There were significant differences observed (p < 0.05). The carotid artery plaques in the two groups were significantly smaller after treatment (P<0.05). There was no significant difference in adverse reactions between the two groups (P > 0.05).

**Conclusion:**

Double doses of atorvastatin combined with febuxostat can effectively reduce uric acid to improve the inflammatory state in patients and reduce carotid plaques without increasing the incidence of adverse reactions.

## INTRODUCTION

Gout is a group of diseases caused by a purine metabolism disorder that is closely related to hyperuricemia (HUA). Studies have shown that the prevalence of HUA in China is 12.08 %,^1^ which is significantly higher than it was 30 years ago, and that HUA is an independent risk factor for the occurrence and development of cardiovascular and cerebrovascular diseases, endocrine diseases and chronic kidney diseases.^2^ It has been reported that patients with gout continue to have micro-inflammation in their bodies even during the intermission period between attacks, and chronic low-grade inflammation is the basis of metabolic syndromes, such as obesity, hyperlipidemia, and atherosclerosis.^3^ A large number of studies have shown that rheumatoid arthritis, which is also a rheumatic immune disease, has a significantly increased risk of carotid plaque formation and cardiovascular events.^4^,^5^ Early treatment with statins can effectively improve lipid metabolism and inflammation indexes and reduce mortality. However, there are relatively few studies on gout complicated with carotid plaque. The purpose of this study was to provide more evidence to guide clinical treatment.

## METHODS

Seventy patients with gout were admitted to our hospital from January 2014 to June 2017, including 46 males and 24 females. PASS software was used to calculate the sample size. A randomized, double-blind approach was used to divide the participants into the treatment group (35 cases, male (24 cases) and female (11 cases)) and the control group (35 cases, male (22 cases) and female (13 cases). There were no significant differences in general data between the two groups (P > 0.05), as shown in Table-I.

**Table-I:** Comparison of General Data of the Two Groups (X̄±s).

Group	n	Male/Female	Age
Treatment group	35	24/11	47.2±6.5
Control group	35	22/13	48.6±7.1

The study was approved by the Institutional Ethics Committee of Affiliated Hospital of Hebei University (No. HDFY-LL-2020-090; date:23-07-2020), and written informed consent was obtained from all participants.

All patients met the 1981 American College of Rheumatology (ACR) gout classification criteria.^3^ (1) The articular fluid was confirmed to contain urate crystals possessing characteristic features, or (2) Confirmation of other markers by chemical methods or polarization microscopy, or (3) Six out of the following 12 findings: acute arthritis attacks occurring more than once; the inflammatory reaction peaked within one day; the onset of monoarthritis; visible joint redness; pain or swelling of the first metatarsophalangeal joint; an attack of the unilateral first metatarsophalangeal; the onset of unilateral tarsal arthritis; presence of a tophus; hyperuricemia; asymmetrical intra-articular swelling (confirmed by X-ray); subcortical cyst without erosion (confirmed by X-ray); and negative microbial culture from synovial fluid at the onset of arthritis.

### Inclusion criteria

Patients between ages 18-65 years were initially treated for gout in the intermittent stage but had not been treated with hyaluronic acid medication, as well as if their uric acid levels were ≥ 480 µmol/l and their course of disease was >2 weeks. The patients were informed about the study and signed a consent form.

### Exclusion criteria

(1) Patients with acute gout onset. (2) Patients receiving theophylline and other drugs to reduce uric acid salt. (3) Patients with cardiovascular and cerebrovascular diseases, endocrine diseases and neuropsychiatric diseases. (4) Patients who had an allergic condition allergy to febuxostat or atorvastatin or other contraindications. (5) Pregnant and lactating women.

### Treatment group

Patients received febuxostat 40 mg once per day and atorvastatin 40 mg once per day. Control group: Patients received febuxostat 40 mg/day and atorvastatin 20 mg/day. The course of treatment was 90 days.

### Observation indicators

The patients’ TNF-α, IL-1β, and CRP levels were examined, as well as their ESRs and carotid plaques. TNF-α and IL-1β were detected by ELISA, and the erythrocyte sedimentation rate (ESR) was detected by Westergren’s method. C-reactive protein (CRP) was detected using a Beckman AU5800 analyzer and the supplier’s original reagent. Carotid plaques were detected by GE S8 ultrasound. SPSS 19.0 statistical software was used. Statistical significance was determined for the differences between the two groups by t test (p < 0.05).

## RESULTS

After 90 days of treatment, serum TNF-α, IL-1β, and CRP levels, as well as HUA and total cholesterol (TC), decreased in both groups after treatment. There were significant differences observed (p < 0.05). Table-II. Changes in carotid plaque: The carotid artery plaques in the two groups were significantly smaller after treatment (P<0.05). Table-III. Adverse reactions: There was no significant difference in adverse reactions between the two groups (P > 0.05). Table-IV.

**Table-II:** Changes in TNF-α, IL-1β, CRP, HUA and TC in the two groups before and after treatment (X̄±s, n=30).

Group	Time	TNF-α (ng/ml)	IL-1β (ng/ml)	CR (mg/L)	HUA (µmol/L)	TC (mmol/L)
Control group	Before treatment	40.83±9.24	3.71±1.62	15.8±6.2	579±72	6.36±1.28
	After treatment	25.63±5.33Δ	3.12±0.96Δ	11.2±3.5Δ	318±49	3.69±0.94Δ
Treatment group	Before treatment	42.41±10.82	3.58±1.86	14.9±5.5	588±67	6.55±1.19
	After treatment	22.79±4.51*Δ	2.87±1.05*Δ	9.6±2.8*Δ	309±41	2.92±0.87Δ

Compared with the same group before treatment,

Δ p < 0.05; compared with the control group

* p < 0.05, * * p > 0.05

**Table-III:** Color Doppler ultrasound of carotid artery plaques before and after treatment in the two groups (X̄±s, n=30).

Group	Time	Patch size (cm^2^)	Patch thickness (mm)	Number of plaques (number)
Control group	Before treatment	0.081±0.026	2.72±0.73	3.72±0.89
	After treatment	0.052±0.021Δ	1.91±0.87Δ	2.71±0.71Δ
Treatment group	Before treatment	0.079±0.030	2.81±0.86	3.61±0.80
	After treatment	0.041±0.017*Δ	1.47±0.75*Δ	2.36±0.68*Δ

**Table-IV:** Color Doppler ultrasound of carotid artery plaque before and after treatment in the two groups (X̄±s, n=30).

Group	Nausea	Headache	Vertigo	Total adverse reactions
Control group	2 (5.7)	1 (2.9)	1 (2.9)	4 (11.4)
Treatment group	1 (2.9)	2 (5.7)	2 (5.7)	5 (14.3)

## DISCUSSION

Gout is a type of acute and chronic inflammation and tissue damage caused by the deposition of monosodium urate in bone joints, kidneys and subcutaneous areas, and it is directly related to hyperuricemia caused by purine metabolic disorder and/or reduced uric acid excretion.^6^ During an acute attack, the main manifestations were inflammation, joint swelling, heat and pain and were usually relieved spontaneously within two weeks. After that, the patient has entered the intermission period between attacks, which is characterized by asymptomatic hyperuricemia. Previous reports have shown that there remains a slight chronic inflammatory state in the patient during the intermission period. It is presumed that uric acid plays an inflammatory role through the crystallization of monosodium urate (MSU) or soluble factors. Some studies have shown that MSU can stimulate the expression of TLR4, NLRP3, TNF-α, and IL-1β.^7^ On the other hand, dissolved uric acid can enter cells and activate MAP kinase (P38 and ERK), which further activates NF-κB, and induce the expression of inflammatory mediators such as MCP-1 and CRP.^8^ Among the implicated factors, TNF-α, IL-1β and CRP are important components of the chronic inflammatory environment.

Tumor necrosis factor-α (TNF-α) is an early-discovered proinflammatory cytokine, and its biological effects have been studied extensively. It is mainly produced by activated mononuclear macrophages and partially produced by T cells. It can promote lymphocyte activation and fibroblast proliferation and has effects that regulate immunity and mediate the inflammatory response. In addition, it also has effects on cytokines, chemokines, prostaglandins and metalloproteases and plays an important pathological role in various autoimmune diseases.^9^ Its role in the course of gout is relatively clear. TNF-α is the main inflammatory mediator in the body. Atherosclerosis is also, by nature, an inflammatory vascular disease. Therefore, in the early stages of atherosclerosis, TNF-α may get involved in the development of atherosclerotic plaques by destroying the integrity of vascular endothelial structure and function, transcription of the SIS protooncogene, proliferation of vascular smooth muscle cells, and the balance of coagulation and anticoagulation systems, etc.^10^-^11^ At the same time, TNF-α can also activate the cytokine system, thereby activating the systemic inflammatory response and accelerating the formation of atherosclerosis.^12^ A considerable number of studies have shown that TNF-α is involved in the process of inflammation and development of atherosclerosis and plays a key role in promoting both.^13^,^14^

In 2006, Martinon and his students proposed that IL-1β and the NALP3 inflammasome are key factors in the inflammatory response.^15^ After the NLRP3 inflammasome is activated in gout patients, inactive IL-1β precursors can be cleaved into mature IL-1β, further amplifying the inflammatory response of the body. At the same time, IL-1β is an important inflammatory factor in the occurrence and development of various metabolic diseases.^16^ Inflammation usually plays a very important role in the pathogenesis of various chronic diseases or states. For example, studies on atherosclerosis have found that cholesterol crystals can produce a large amount of IL-1β^17^,^18^ by activating the NALP3 inflammasome. Geng et al confirmed that IL-1β is an atherogenic cytokine and can promote apoptosis of normal vascular smooth muscle cells; inflammation plays an important role in gout and atherosclerosis.^19^ The two are intrinsically linked.

Increasing data show that uric acid is involved in many aspects of the occurrence, progression and deterioration of atherosclerosis. Therefore, it is very important to reduce uric acid while improving atherosclerosis and reducing inflammatory reactions. Febuxostat is a new xanthine oxidase inhibitor that has an obvious inhibitory effect on the oxidation and reduction forms of the enzyme and can noticeably reduce the level of uric acid. At the same time, it can also improve the inflammatory state of the body. Atorvastatin is an HMG-CoA reductase inhibitor that has a significant lipid-lowering effect along with low toxicity and relative safety. In recent years, atorvastatin has also been found to be anti-inflammatory and anti-atherosclerotic. It can effectively reduce the levels of inflammatory factors such as high-sensitivity C-reactive protein and TNF-α in diabetic patients in a short period of time. Several studies have shown that atorvastatin can affect the expression of nuclear factor κB, TLR4 and its downstream signals and MMP-9, inhibit inflammatory reactions and stabilize atherosclerotic plaques in various ways.^20^,^22^

The results showed that TNF-α, IL-1β, CRP and other inflammatory indexes in the treatment group were significantly lower than those in the control group. Atherosclerotic plaque area decreased, indicating that double doses of atorvastatin combined with febuxostat can better reduce inflammatory reactions in patients with gout and carotid atherosclerosis. The curative effect of a double dose of atorvastatin was fully affirmed, and the number of cases of nausea, headache, dizziness and insomnia in the treatment group was not different from that in the control group. These results showed that the double dose of atorvastatin in the test group did not increase the incidence of adverse reactions.

### Limitations of the study

Dietary factors and other metabolic related diseases may have an impact on the results of the study. Therefore, a multi factor analysis will be carried out in the next step to observe the correlation between various factors and the research indicators.

## CONCLUSION

In summary, the double dose of atorvastatin combined with febuxostat in the treatment of patients with gout and carotid atherosclerosis can effectively reduce uric acid, improve the inflammatory state in patients, reduce carotid plaque and have good clinical benefits.

### Authors’ Contributions:

**ZZ &** **MHX:** Designed this study and prepared this manuscript, and are responsible and accountable for the accuracy or integrity of the work.

**LS:** Collected and analyzed clinical data.

**FW:** Significantly revised this manuscript.

**ZZ** **&** **MX:** contribute to this work equally.
